# Discrepancies in dyadic coping: associations with distress and quality of life in couples facing early stage dementia

**DOI:** 10.3389/fpsyg.2023.1056428

**Published:** 2023-05-24

**Authors:** Peter Muijres, Katharina Weitkamp, Guy Bodenmann, Josef Jenewein

**Affiliations:** ^1^University of Zurich, Department of Psychology, Clinical Psychology for Children/Adolescents and Couples/Families, Zurich, Switzerland; ^2^Private Clinic Hohenegg, Meilen, Switzerland; ^3^Department of Psychiatry and Psychotherapy, University of Zurich, Zurich, Switzerland

**Keywords:** dyadic coping, dementia, equity, reciprocity, congruence

## Abstract

**Objectives:**

Due to an aging population, the number of persons living with dementia (PWDs) is increasing worldwide. Romantic partners, as informal caregivers (IC) of PWDs, are often adopting additional tasks. The concept of dyadic coping (DC) addresses how couples cope with stress together. For dyadic coping to be successful, efforts of both partners should be equal. The current study examines how discrepancies in PWDs and ICs perspectives on DC relate to distress and quality of life in each partner within couples facing early stage dementia (ESD).

**Methods:**

A total of 37 mixed-sex couples including one partner with ESD completed self-report questionnaires. Discrepancies in reciprocity (comparing provided or received levels of DC between partners), equity (each partner balancing own levels received and provided), and congruence (the agreement about levels of DC exchanged between partners) and their covariation with distress and quality of life (QoL) of each partner were measured.

**Results:**

Both partners indicated a discrepancy in reciprocity: PWDs reported receiving more DC than ICs reported receiving, which was associated with higher QoL in PWDs and lower QoL in ICs. Inequities were found in ICs only, who reported receiving less DC, than providing. No relation between inequities and distress or QoL was found. ICs reported more incongruencies than PWDs did, which was associated with higher QoL and less depression in partners.

**Discussion:**

A redivision of tasks and roles in the early stage of dementia is associated with different experiences and views between partners. Whereas ICs take over most household and care tasks within the couple, their effort was considered less helpful by PWDs than by ICs. A high care burden is associated with a compromised quality of ICs’ social life and living conditions. The clinical implications of the results are discussed.

## Introduction

Over 50 million people are estimated to be living with dementia worldwide (Alzheimer’s Disease International, 2019), making dementia among the biggest public health concerns today. According to the Federal Office of Public Health (Federal Office of Public Health (FOPH), 2020), dementia prevalence rates increase steeply above the age of 65 years old and concern approximately 12% of individuals of 80–84 years old. In Switzerland today, 110,000 people have developed dementia. This number is expected to reach 190,000 by 2030 (Federal Office of Public Health (FOPH), 2020).

At least half of persons living with dementia (PWDs) are living at home (Hallauer, 2004; Romero, 2011; Chester et al., 2018; Clarkson et al., 2021). The partner is the primary coping resource for couples (Manne and Badr, 2008; Badr and Acitelli, 2017). Many older people prefer the familiarity of communication with a partner (Carstensen, 1992). Health issues, retirement, and family and friends passing away account for a decreasing social network and make the partner even more important as a source of social support. As the illness progresses, so does the dependence of PWDs on the support of partners, providing informal care (Gagnon et al., 1999).

Dementia is a neurodegenerative disorder featuring increasing difficulties in performance in six domains: memory, orientation, judgment and problem-solving, community affairs, home and hobbies, and personal care (Morris, 1997). PWDs become more dependent when the symptoms manifest themselves, and an illness-related burden increasingly takes its toll on both members of the dyad (Etters et al., 2008; Häusler et al., 2016). In PWDs, a depressed mood, psychological distress and a deteriorating health, accounts for much of the burden (Pinquart and Sörensen, 2003; Häusler et al., 2016). For informal caregivers (ICs), the burden involves the overall physical, psychological, emotional, and financial toll of providing care (Dang et al., 2008; Stenberg et al., 2010; Feinberg et al., 2011; Chiao et al., 2015). Caregiving partners having to cope with illness-related stresses and simultaneously in need of support themselves occupy a dual role (Revenson, 1994). In the literature, high levels of stress in ICs, exceeding stress in PWDs, are well documented (Revenson, 1994; Campbell, 2009; Häusler et al., 2016; Lee et al., 2019). Although both partners suffer from a physical or mental illness of one partner, they also possess resources to cope with the stress together (Leuchtmann and Bodenmann, 2017).

Couples coping with illness-related stress, who do not discuss their differences in views, will grow further apart (Acitelli and Badr, 2005). Depending on the stressor at stake, the coping resources of both partners are activated in dyadic coping (DC) in an attempt to stabilize the partner to reduce one’s own stress and maintain or restore a state of homeostasis among both partners, within the couple as a unit and in regard to the social environment (Bodenmann, 1995). DC affects the extent to which caregiver burden is translated into caregiver stress (Häusler et al., 2016). Without the means to communicate, share, and cope with stress as a couple, both PWDs and ICs are at increased risk of anxiety, depression, and sleeping disorders in the previous study (Revenson and Hoyt, 2016).

The interdependence between PWDs and ICs advocates addressing stress regulation at a dyadic level (Van’t Leven et al., 2013) and in an early stage of dementia (Wuttke-Linnemann et al., 2019). Particularly at the onset, PWDs and ICs often feel overwhelmed by current and future illness-related losses and care-related challenges (Clare, 2003; Harman and Clare, 2006; Steeman et al., 2006). Given the progressive nature of dementia, PWDs are best able to voice their care-related values and preferences, when symptoms in PWDs are still mild (Whitlatch et al., 2005; Steeman et al., 2006; Orsulic-Jeras et al., 2019). When information about care-related values and preferences is not expressed and discussed by both partners in an early stage of dementia, then an unfavorable pattern of coping with dyadic stress is more likely to develop in later stages of dementia, when the window of change has closed (Maslow, 2013; Orsulic-Jeras et al., 2019).

The concept of DC is based on a systemic-transactional view of stress and coping among couples (STM; Bodenmann, 2005; Bodenmann et al., 2016). DC describes the interplay between partners in a close relationship where one is signaling stress verbally, paraverbally or non-verbally and the other is verbally and/or non-verbally reacting to those signals (Bodenmann, 1995, 2005; Ledermann et al., 2010).

**Figure 1:**
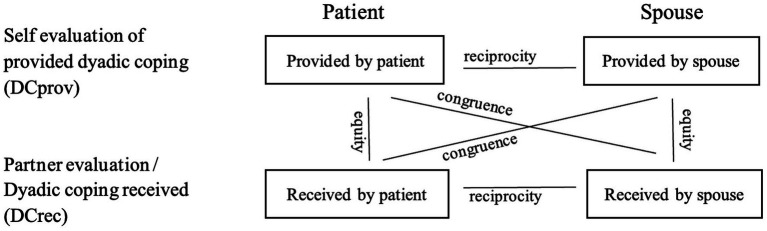
Reciprocity, equity, and congruence in relation with the dyadic coping exchanged between patients and partners (Bodenmann, 2008, p. 28).

Discrepancies in DC reflect diverging perceptions on and differences in DC exchanged within the couple. A discrepancy in reciprocity entails a comparison between the evaluations of both partners regarding their own behaviors and the evaluation of behaviors of the partner (Bodenmann, 2008). Reciprocity addresses the question “Which partner reports to provide (or receive) more DC?” and is also referred to as “similarity index” (Kenny and Acitelli, 2001). Equity in DC describes the perceived individual balance between levels of DC received from and provided to other partner and has also been called “fairness” (Meier et al., 2020) or “assumed agreement” (Kenny and Acitelli, 1989; Iafrate et al., 2012). Congruence in DC describes the agreement about the DC levels provided by one and received by the other partner and has been also referred to as “understanding” (Kenny and Acitelli, 2001), “accuracy” (Acitelli et al., 2001), or “consistency index” (Felser, 2003). An overview of the three types of discrepancies is shown in Figure 1.

Reciprocity or similarity about mutual contributions within the relationship is a critical dimension for marital relationship quality (Deal et al., 1992; Acitelli et al., 2001; Meier et al., 2020, 2021). Endangering the shared experience of coping as a couple together, and associations with adjustment problems and distress have been found for inequity (Acitelli and Badr, 2005; Gmelch and Bodenmann, 2007; Meier et al., 2020, 2021) and incongruence in DC (Kayser et al., 2007; Iafrate et al., 2012; Meier et al., 2021). Effective DC is associated with equal efforts of both partners (Meier et al., 2020). Higher differences in views of DC exchanged are generally associated with more distress in both partners.

Despite its growing clinical implications for PWDs’ adjustment and mental health, no research to date has studied yet how discrepancies in perceptions of exchanged levels of DC differ in couples facing ESD and how these differences correlate with distress and QoL.

## Method

### The current study

The current study was part of a larger project on the feasibility, acceptance, and benefits of dignity therapy in people with ESD and their relatives (Jenewein et al., 2021). Our first goal was to analyze whether views of DC between PWL and ICs differed significantly. Our second goal was to analyze whether discrepancies found were associated with higher distress and lower quality of life (QoL) in individual members of the dyads. Discrepancies and their correlations with distress and QoL were addressed first in reciprocity, then in equity, and in congruency at last.

### Participants

PWDs were recruited at the University Geriatric Outpatient-Center Waid, Switzerland, between March 2019 and October 2020. Inclusion criteria were adult persons with a diagnosis of very mild or mild dementia corresponding with a Clinical Dementia Rating of between 0.5 and 1.5 (Morris, 1997) who were in a close and committed, heterosexual relationship with an IC unaffected by chronic health conditions. Exclusion criteria were PWDs or ICs having insufficient knowledge of the German language and couples including ICs with a chronic illness themselves.

Among the 37 heterosexual couples, 36 were married (97.3%) for an average duration of 46.9 years (range 5.0–67.0, *SD* = 13.90). Of the 37 PWDs, 23 persons were men (62%) and 14 persons were women (38%). The average age of PWDs was 78.8 years old (range 63–89, *SD* = 5.86) and of ICs was 76.8 years (range 59–90, *SD* = 7.22). Table 1 shows basic sociodemographic data.

**Table 1:** Participants’ basic demographics.

		PWDs (*n* = 37)		ICs (*n* = 37)	
		*n*	%	*n*	%
Gender	Female	14	37.8	23	62.2
	Male	23	62.2	14	37.8
Occupation	Part-time employed	2	5.4	8	21.6
	Full-time employed	0	0	7	18.9
	Pensioner	33	89.2	19	51.4
	Housekeeper	2	5.4	3	8.1
Relationship status	Married	36	97.3	36	97.3
	Unmarried	1	2.7	1	2.7
Education level	Compulsory education	4	10.8	3	8.1
	Apprenticeship	14	48.6	23	62.2
	Secondary school	5	13.5	0	0
	Technical college	8	21.6	5	13.5
	University	6	16.2	6	16.2
Religion	Roman catholic	12	32.4	5	13.5
	Protestant	14	48.6	24	64.7
	Christian ‘other’	2	5.4	1	2.7
	Islam	0	0	0	0
	Other	2	5.4	1	2.7
	No religious affiliation	8	21.6	6	16.2

Mean age (*SD*) in years is 78.8 (5.86) in PWDs and 76.8 (7.32) in ICs (*n* = 37). Mean relationship duration (*SD*) of couples in years is 46.9 (13.90). PWDs = persons living with dementia; ICs = caregiving partners.

### Procedure

The consulting physicians at the study site made eligible PWDs aware of the study. With their permission, contact details of eligible PWDs were passed on to the study coordinator. The study coordinator then first contacted them by telephone and informed them about the study and the use of questionnaires. Couples, interested in participating, were sent a study information folder, and a personal information visit at home was planned.

The information visit provided ample opportunity to ensure that both PWDs and ICs were comprehensively informed about all aspects of the study procedure. Both PWDs and ICs signed an informed consent form before completing the baseline assessment, including the DCI, when they were in a romantic relationship together. During data collection, the study coordinator remained available for assistance and verified an accurate understanding and completion of items in case of doubt.

### Measures

The *Dyadic Coping Inventory* was used to assess DC (DCI; Bodenmann, 2008). The DCI is a 37-item self-report instrument measuring DC behavior on a five-point Likert-type scale (1 = hardly ever and 5 = very often). Both PWDs and ICs filled out the DCI. Each participant rated the levels of DC they provided (DCprov) to their partner, as well as the levels they received from their partner (DCrec). The main subscales of the DCI were used in this study.

Stress communication (SC) represents the ability of a stressed person to communicate his or her stress to their partner and ask for support (e.g., “I ask my partner to do things for when I have too much to do”). Supportive DC (SDC) describes one partner providing problem-and/or emotion-oriented support to assist the other in his or her coping efforts (e.g., “I show empathy and understanding towards my partner”). Delegated DC (DDC) involves efforts of the other partner to relief the stressed partner by taking over his or her tasks and responsibilities (e.g., “When my partner feels he/she has too much to do, I help him/her out”). Negative DC (NDC) includes hostile, ambivalent, or superficial actions or words (e.g., “I do not take my partner’s stress seriously”). Common dyadic coping (CDC) describes both partners experiencing stress and their joint effort to cope with it (e.g., “We engage in a serious discussion about the problem and think through what has to be done”; Bodenmann, 1995, 2005; Bodenmann et al., 2016, 2018).

By completing the DCI, each participant generates multiple subscales, of which 10 dyadic subscales were used in the current study. The 10 dyadic subscales for PWDs include the stress communication that PWDs received from their partners (SCrec_pwd_) and the stress that PWDs provided to (i.e., expressed toward) their partners (SCprov_pwd_); furthermore, the supportive dyadic coping that PWDs received from their partners (SDCrec_pwd_) and provided to their partners (SDCprov_pwd_); e.g., the delegated dyadic coping that the patient received from (DDCrec_pwd_) and provided to their partner (DDCprov_pwd_); and the negative dyadic coping the patient received from (NDCrec_pwd_) and provided to their partner (NDCprov_pwd_); lastly, the total dyadic coping the patient received from (DCrec_pwd_) and provided to the other partner (DCprov_pwd_). By independently completing the DCI, the patient and the partner yield scores that can be compared to each other.

In addition to the means of separate subscales, additional information about the relationship is revealed by the mean difference between subscales, and the use of discrepancy measures is therefore advocated (Gmelch and Bodenmann, 2007). Discrepancies were indexed by subtracting the averages of DCprov from DCrec. Positive differences represent an overbenefit: one has received more than one provided. Should one report having received less than one provided, then the negative outcome reflects an underbenefit (Meier et al., 2020, 2021).

Significant differences in equity, reciprocity, and congruence between or within partners were first used to compute discrepancy indexes that were then used to calculate the correlations with the outcome measures of PWDs and ICs with. To calculate reciprocity index for stress communication, for example, in SCprov, mean partner levels were always subtracted from mean patient levels of DC (i.e., RPR_SCprov = SCprov_pwd_ – SCprov_IC_). A negative mean difference thus indicates significantly higher IC levels than PWD levels of SCprov. The equity and reciprocity indexes were consistently calculated by subtracting the provided levels from the received levels of DC (e.g., EQ_DC = DCrec – DCprov) for equity and congruence indexes and for congruence indexes, and by subtracting IC levels from PWD levels (e.g., CGR_DCrec_pwd_ = DCrec_pwd_ – DCprov_IC_). Figure 1 illustrates how the discrepancies are composed. The psychometric properties of the DCI were considered as good. Internal consistency (Cronbach’s alpha) of the DCI ranged between 0.71 and 0.93.

*The Hospital Anxiety and Depression Scale (HADS)* is a 14-item self-report questionnaire measuring states of anxiety (HADS-A) and depression (HADS-D) on a four-point response scale (e.g., 0 = not at all and 3 = very often). The HADS was originally developed from a study in the outpatient clinic of a general medical hospital (Zigmond and Snaith, 1983). In the current study, Cronbach’s alpha for the HADS total score was 0.87.

*The World Health Organization Quality of Life Questionnaire (WHOQOL-BREF)* is a widely used 26-item self-report instrument, based on a five-point Likert Scale and not related to a specific disease. The WHOQOL-BREF consists of five subscales, which are physical health, psychological health, social relationships, environment, and global, the latter subscale reflecting overall quality of life (QoL) and general health (Angermeyer et al., 2000). In the current study, Cronbach’s alpha ranged from 0.58 (social domain) to 0.81 (psychological domain).

### Data analyses

#### Identifying significant differences in reciprocity, equity, and congruence

Before composing and using a discrepancy index, the mean difference was calculated between the two subscales concerned. Considering the normal distribution and dyadic structure of the data, analyses were made using paired-samples *t*-tests. When the variables differed on a 95% confidence interval (*p* < 0.05), then the discrepancy between the mean scores compared was significant and used for further analysis. When no significant difference was found, an association between a discrepancy and distress or QoL could be ruled out and the discrepancy index excluded from further analysis.

The next step consisted of calculating correlations between significant discrepancy indexes and the outcome measures. Pearson’s correlations were used to calculate correlations between each discrepancy index related to a significant difference and the seven outcome measures: anxiety, depression, and five domains of QoL, in both PWDs and ICs. We calculated a *post hoc* power analysis for the sample size of *n* = 37 for correlations assuming a medium effect. The test power was 0.59.

However, since education level and severity of disease were found to relate to differences in anxiety and depression, as well as to QoL (Lampert and Kroll, 2009), Pearson’s correlations were calculated to investigate that possibility. Since no significant correlations were found, education level and severity of disease were assumed not to account for associations found between discrepancy indexes and anxiety, depression, and QoL and were thus not controlled for.

Given the dyadic nature of the data, the strength of the relationship between discrepancy indexes and the outcome measures was established using correlation coefficients and *p*-values. Based on Cohen (1988, 1992), the following criteria were used to assess the effect sizes of correlation coefficients: *r* = 0.1 (small effect), *r* = 0.3 (medium effect), and *r* = 0.5 (large effect). IBM SPSS statistics software version 27 was used for the data analyses.

## Results

The mean scores of the DC scale were first calculated and displayed in Table 2.

**Table 2:** Means of stress communication and dyadic coping variables.

	PWD (*n* = 37)		IC (*n* = 37)	
	*M*	*SD*	*M*	*SD*
SCrec	3.03	0.85	3.21	0.89
SCprov	3.28	0.95	2.79	0.77
SDCrec	3.69	0.78	2.83	1.24
SDCprov	3.59	0.83	3.83	0.66
DDCrec	3.96	0.88	2.74	1.19
DDCprov	3.62	0.99	4.39	0.64
NDCrec	2.01	0.72	1.97	0.84
NDCprov	2.22	0.81	1.90	0.77
TDCrec	3.63	0.51	3.24	0.75
TDCprov	3.56	0.58	3.70	0.48

SC = stress communication; DC = dyadic coping; SDC = supportive dyadic coping; DDC = delegated dyadic coping; NDC = negative dyadic coping; TDC = total dyadic coping; rec = received; prov = provided.

### Reciprocity in DC

A comparison of PWDs’ and ICs’ reciprocity levels yielded five significant differences, as shown in table. Reports of SCprov were significantly higher in PWDs than in ICs (*t* (36) = 2.75, *p* < 0.01). Reports of PWDs of DDCrec were also higher (*t* (36) = 5.30, *p* < 0.001), and reports of DDCprov were accordingly lower than ICs (*t* (36) = −3.44, *p* < 0.01). PWDs reported receiving significantly more SDC (*t* (36) = 4.24, *p* < 0.01) and TDC (*t* (36) = 3.06, *p* < 0.01) than ICs did. No significant differences between PWDs and ICs were found for SCrec, NDCrec, NDCprov, or TDCprov (see Table 3).

**Table 3:** Discrepancies in reciprocity in stress communication and dyadic coping between PWDs and ICs.

Reciprocity measure	*M*	*SD*	*t*	*p*
SCprov	0.49	1.08	2.75	0.009**
SCrec	−0.24	1.09	−1.36	0.182
DDCprov	−0.77	1.36	−3.44	0.001**
NDCprov	0.32	1.11	1.73	0.092
TDCprov	−0.14	0.72	−1.17	0.252
SCrec	−0.18	1.40	−0.77	0.449
SDCrec	0.85	1.23	4.24	0.001**
DDCrec	1.21	1.40	5.30	**<0.001**
NDCrec	0.04	0.91	0.27	0.789
TDCrec	0.34	0.68	3.06	0.004**

SC = stress communication; DC = dyadic coping; PWDs = persons living with dementia; ICs = caregiving partners; SCprov = stress communication provided; SCrec = stress communication received; DDCprov = delegated dyadic coping provided; NDCprov = negative dyadic coping provided; TDCprov = total dyadic coping provided; SCrec = stress communication received; SDCrec = supportive dyadic coping received; DDCrec = delegated dyadic coping received; NDCrec = negative dyadic coping received; TDCrec = total dyadic coping received. *p < 0.05, **p < 0.01, and ***p < 0.001 (*n* = 37).

#### Correlations between reciprocity indexes and distress and QoL in PWDs and ICs

No significant correlations between the five significant reciprocity indexes, anxiety, and depression were found within couples.

A total of five significant correlations were found between the discrepancies in reciprocity and QoL: one for PWDs and four for ICs. The results are shown in Table 4. In terms of reciprocity, ICs reported having provided more DDC than PWDs did, which correlated positively with PWDs’ psychological QoL (*r* = 0.33, *p* < 0.05) and negatively with ICs’ social QoL (*r* = −0.35, *p* < 0.05). Significant negative correlations were found between the discrepancies in SDCrec and environment-related QoL in ICs (*r* = −0.32, *p* < 0.05), as well as between TDCrec and the environment-related (*r* = −0.45, *p* < 0.01), and global (*r* = −0.37, *p* < 0.05) domains of QOL in ICs.

**Table 4:** Discrepancies in equity in stress communication and dyadic coping according to PWDs and ICs.

	Equity measures	*M*	*SD*	*t*	*p*
PWD	SC	−0.24	1.15	−1.29	0.207
	SDC	0.10	0.76	0.78	0.444
	DDC	0.34	1.23	1.67	0.104
	NDC	−0.20	0.65	−1.91	0.064
	TDC	0.07	0.46	0.89	0.381
IC	SC	0.42	1.10	2.31	0.027*
	SDC	−1.00	1.03	−5.91	0.000***
	DDC	−1.65	1.49	−6.75	**<0.001**
	NDC	0.07	0.70	0.64	0.525
	TDC	−0.46	0.64	−4.35	< 0.001***

PWDs = persons living with dementia; ICs = caregiving partners; DC = dyadic coping; SC = stress communication; SDC = supportive dyadic coping; DDC = delegated dyadic coping; NDC = negative dyadic coping; TDC = total dyadic coping. Significant differences are printed in bold and indexed (*n*_tot_ = 37). *p < 0.05; **p < 0.01, ***p < 0.001 (*n* = 37).

### Equity in DC

In total, four significant discrepancies were found for equity, referring to the subjective “fairness” between levels of DCprov and DCrec, all accounted for by ICs and shown in Table 4.

The results show that, according to ICs, the level of stress signals they received is significantly higher than the level they provided (i.e., expressed) themselves (*t* (36) = 2.31, *p* < 0.05). ICs also reported to receive a significantly lower level of SDC than the level they provided to PWDs (*t* (36) = −5.91, *p* < 0.001). Furthermore, similar underbenefits for ICs applied to DDC and TDC: ICs reported receiving significantly less DDC (*t* (36) = −6.75, *p* < 0.001) and less total DC from PWDs (*t* (36) = −4.35, *p* < 0.001) than they were providing themselves. Whereas ICs reported four significant disbalances between levels of DCrec and DCprov, disbalances were reported by PWDs.

#### Correlations between equity indexes and distress and QoL in ICs

None of the four equity indexes found in ICs correlated significantly with any of the outcome measures.

### Congruence in DC

A total of four significant discrepancies, displayed in Table 5, were found in the analysis of congruence within couples concerning DC levels transferred. The reported level of DDCrec was lower than the other partner reported having provided, according to both PWDs [*t* (36) = −2.51, *p* = 0.017] and ICs [*t* (36) = −6.75, *p* < 0.001]. ICs reported receiving less SDC than PWDs reported having provided to IC [*t* (36) = −3.39, *p* < 0.01]. Lastly, ICs reported having received less total DC than PWDs reported having provided [*t* (36) = −2.56, *p* < 0.05].

**Table 5:** Discrepancies in congruence in stress communication and dyadic coping exchanged between PWDs and ICs.

Congruence between	Congruence measure	*M*	*SD*	*t*	*p*
PWD reported DCrec vs. IC reported DCprov	Stress communication	0.24	0.97	1.53	0.134
	Supportive dyadic coping	−0.15	0.86	−1.03	0.309
	Delegated dyadic coping	−0.43	1.05	−2.51	0.017*
	Negative dyadic coping	0.11	0.96	0.73	0.470
	Total dyadic coping	−0.07	0.58	−0.76	0.454
PWD reported DCrec vs. IC reported DCprov	Stress communication	−0.07	1.24	−0.33	0.741
	Supportive dyadic coping	−0.76	1.36	−3.39	0.002**
	Delegated dyadic coping	−1.65	1.49	−6.75	**<0.001**
	Negative dyadic coping	−0.24	1.10	−1.34	0.188
	Total dyadic coping	−0.32	0.77	−2.56	0.015*

SC = stress communication; DCrec = dyadic coping received; DCprov = dyadic coping provided; SC = stress communication; SDC = supportive dyadic coping; DDC = delegated dyadic coping; NDC = negative dyadic coping; TDC = total dyadic coping. Significant differences are printed in bold. *p < 0.05; **p < 0.01, ***p < 0.001 (*n* = 37).

#### Correlations between congruence indexes and distress and QoL in PWDs and ICs

No significant correlation was found with either distress or QoL in PWDs, yet, when correlations were calculated between congruence indexes and ICs related outcome measures, seven significant results were found.

Two positive significant correlations were found incongruence in the level of SDC exchanged (ICs received less than PWDs provided), the environment-related domain (*r* = 0.38, *p* < 0.05), and the global domain of QoL in ICs (*r* = 0.44, *p* < 0.01). Positive correlations were found between the incongruencies in DDC (ICs reported receiving less DDC than PWDs reported having provided) and global QoL in ICs (*r* = 0.41, *p* < 0.05). Concerning TDC (ICs reporting lower TDCrec than PWDs reported having provided), four significant correlations with outcome measures in ICs were found. One negative correlation with depression (*r* = −0.39, *p* < 0.05) was found and three positive correlations between TDC and QoL in the psychological domain (*r* = 0.43, *p* < 0.01), the environmental domain (*r* = 0.39, *p* < 0.05), and the global domain (*r* = 0.50, *p* < 0.01).

## Discussion

This article explored discrepancies in reciprocity, equity, and congruence of DC and their associations with anxiety, depression, and QoL. Our expectations that higher discrepancies in DC levels exchanged would relate to more distress and lower QoL in both PWDs and ICs were partially confirmed.

### Reciprocity in DC between PWDs and ICs

In spite of our expectations of higher stress levels in ICs, than in PWDs, PWDs reported to communicate more stress than ICs reported (i.e., a discrepancy in reciprocity) and ICs reported lower levels of SC received than they expressed (inequity). However, an illness-related functional regression helps explain that PWDs effectively need more support and may thus be soliciting for it in verbal, and para-and non-verbal ways as well by expressing their distress. ICs may need and even want less help from PWDs and, accordingly, express their stress less openly. Reasons to inhibit stress and not invite support from PWDs may be several, such as saving PWDs from additional stress. Hiding negative emotions to spare the patient additional distress is known as “protective buffering” and described by Coyne and Smith (1991). ICs may also expect unuseful or even counterproductive support, possibly even increasing to their stress.

A functional decline and dependency also provide an explanation for PWDs reporting to receive more supportive, delegated and total DC, which was all in line of our expectations. PWDs need more instructions and guidance when performing tasks, and tasks and responsibilities are increasingly taken over by the ICs, raising the total dyadic care burden for ICs, as the illness progresses. Having an IC taking over tasks and responsibilities may imply less task-related stress and confrontation with adverse ESD-related consequences for PWDs. A poor social life and living conditions of ICs were associated with an uneven work distribution. The high care burden and asymmetrical stress communication within the couple may coincide with a high demand in ICs for external social support, such as friend, family, or professional support. However, a loss of cognitive and communicative acuity may make social interaction more demanding less attractive for PWDs, which may lead to social withdrawal and a shrinking social network that affects both members of a couple.

### Equity in perceptions of DC in PWDs and ICs

Inherent to their role, care providers are asking for less help than the people they care for, e.g., by providing emotional support or taking over tasks (DDC). PWDs, being less capable of completing tasks independently, require additional instructions and guidance throughout the process. In addition, tasks and responsibilities are increasingly being taken over by ICs, thus already raising the total dyadic care burden for them in the early stage of dementia. Given the marked differences in equity in DC, ICs appear to hold the support they receive in lower regard than the support they receive. The disagreement within the couple about the effective extent of support exchanged confirms a different appreciation of DC according to each partner.

### Congruence in perceptions of DC between PWDs and ICs

Disagreements within couples about the levels of DC exchanged suggest additional information about the discrepancies found in reciprocity and equity. Interestingly, both PWDs and ICs reported having helped out by taking over tasks more, than their partner agreed with. While care providers may feel inclined to regard all their support as a necessity, PWDs may feel underestimated in their capabilities and underchallenged. Receiving miscarried support attempts may bolster feelings of inadequacy, dependency and indebtedness, and lower self-esteem (Rafaeli and Gleason, 2009). ICs may show overprotective or controlling behaviors toward PWDs, such as closely monitoring or offering excessive support, when PWDs are completing tasks (Bannon et al., 2021). According to ICs in couples facing young onset dementia, not taking over all tasks completely may actually lead to more sources of stress and frustration (Wawrziczny et al., 2016) or work to supervise, correct, or clean up after PWDs’ miscarried performance (Muijres, 2021, same sample; Wawrziczny et al., 2016). Although associations with distress were hardly found for PWDs or ICs, the unequal distribution of dyadic support and high burden may compromise ICs in their living conditions and social life (Lockeridge and Simpson, 2013; Wawrziczny et al., 2016).

While PWDs may question the necessity of all tasks and responsibilities taken over and away from them, ICs may question to what extent PWDs are able to successfully complete tasks and, as well, to provide emotion-or problem-oriented support as a romantic partner at eye level. The early stage of dementia may present the departure point of diverging views, roles and communication feeding into individual and dyadic stress, that may precipitate a poor adjustment at later stages (Wawrziczny et al., 2016; Bannon et al., 2021) and drive a couple apart (Martin et al., 2009).

Unexpectedly, the congruential underbenefits of ICs were consistently associated with *more* wellbeing in ICs (i.e., less depression and more QoL). Positive IC experiences in spite of diverging views about the illness-related role transition within the couple might be tentatively explained by a distracting effect associated with the uptaking of new tasks and responsibilities. Providing emotional support has been found to have health benefits for ICs and reduce their mortality rates (Brown et al., 2003). When symptoms are still relatively new and (very) mild, ICs may be still full of spirits to provide support and derive a sense of pride and purpose from their new caretaker role, whereas additional demands may still be manageable (Leuchtmann and Bodenmann, 2017). However, interpretations ought to be made with care. The DCI addresses dyadic coping of couples with stress in general. Specific inferences about dementia-related coping by couples should be made tentatively and be informed by results of sound empirical studies mapping the interplay between stressors, coping, adaptation, and wellbeing from each partner’s individual and from dyadic perspectives in different dementia-related stages (e.g., Braun et al., 2009; Martin et al., 2009; Rafaeli and Gleason, 2009; Wawrziczny et al., 2016; Gellert et al., 2018; Bannon et al., 2021; Mittelman et al., 2021).

### Limitations

The current study pioneered into examining discrepancies in DC between PWDs and ICs, in terms of reciprocity, equity, and congruency, and how significant discrepancies found interrelated with distress and QoL in PWDs and ICs. The DCI is an innovative tool allowing for a better understanding of couples’ communication and problem-solving. Juxtaposing discrepancies in DC increased the empirical basis to understand couples’ coping in general and inform the clinical consultation of couples facing ESD.

Several limitations need to be noted. Unfortunately, the study was underpowered. The sample of romantic couples was derived *post hoc* from a larger sample of participant pairs in the dignity therapy study, and the size of the subsample had not been determined in advance. Future research should aim to realize data collection with sufficient power to detect small associations. Then, the results about dyadic coping in couples facing ESD are not generalizable to couples coping with ESD.

Dementia is a disorder associated with a gradual decline in judgment, reflective, and communicative abilities (Örulv and Nikku, 2007). It could be argued that reflective abilities have been too limited in PWDs to warrant the validity and reliability of answers. However, anticipating this risk, inclusion criteria were set to admit only participants with very mild or mild symptoms. During data collection, the study coordinator was available for assistance and verification that items were completed as intended. Internal consistency of the DCI in this study was good, as was the internal and concurrent validity in a study using the DCI on couples facing young onset dementia (Häusler et al., 2016). With no items missing in the final data set, there is no indication that a lack of abilities to interpret and answer items motivated participants to skip questions in this study.

In spite of possible risks for validity and reliability, another limitation is the inclusion of PWDs with early stage of dementia only. Changes and stressors are likely to change over time, when symptoms exacerbate and conditions change. Bannon et al. (2021) found a different pattern of DC in couples shortly after the dementia diagnosis. According to the three-phase model of dyadic adaptation to dementia (Martin et al., 2009), stressors and needs change in a non-linear way and ask for a responsive adaptation of activities and strategies to maximize the autonomy and wellbeing of both partners over the course of illness-related stages. Initial patterns of DC in an early stage may precipitate a poorer adjustment at later stages, and future studies in couples facing more advanced stages of dementia would bolster a better understanding of factors supporting a successful adjustment.

Another limitation is the lack of differentiation between male and female PWDs. Researchers pointed out that gender matters in support provision (Bodenmann et al., 2015). Although a subsample of 14 female PWDs with ICs lacks the statistical power to point out existing effects, future studies with larger samples sizes might want to take the potential role of gender differences into account.

Little empirical research has examined the effect of culture on dyadic appraisal and coping processes surrounding chronic illness (Falconier et al., 2016). As people with different cultural backgrounds differ in their attitudes toward DC, a comprehensive understanding related to PWDs and their ICs would benefit from a more diverse cultural sample.

In spite of several weaknesses, the current study was the first ever to examine discrepancies in DC and the relation with distress and QoL in both PWDs and ICs. Taking a dyadic perspective is a key to understand how caregiving affects wellbeing in both partners and changes the relationship of couples facing dementia (Braun et al., 2009). The results carry forward multiple recommendations for clinical practice. First, discrepancies in dyadic coping illustrate an asymmetrical relationship between the care provider and the care receiver that could also erode intimacy and communication at eye level between romantic partners. Familiarizing the couple with the availability of external support to draw upon may help keep stress at bay and warrant a quality of life. Discussing illness-related changes and needs may grant the redistribution of tasks the experience of “we-ness” and improve perceptions of tasks and responsibilities being redivided. Understanding how the intended support from an IC is received by the patient plays an important role in understanding and supporting the partner’s adjustment (Berg and Upchurch, 2007). Clinical consultation may sensitize ICs when to take over tasks and when not, or in what way tasks could fit to PWDs’ capabilities and reduce dependency related stress compromising individual and wellbeing within the couple.

An examination of the relation between DC, distress, and QoL would benefit from a repetition of this study with couples facing more advanced stages of dementia. A replication of this study with a bigger sample may point out significant differences. With a larger and more diverse sample, gender and cultural differences could be taken into account. The use of qualitative interviews and/or additional questionnaires concerning illness-related coping in studies with a longitudinal design could elucidate how couples’ coping may change as illness conditions change over time. The current study considered distress and QoL measures in individual partners. Future studies may wish to consider the inclusion of dyadic outcome measures, including relationship satisfaction, to enable a better understanding of couples’ coping with dementia.

## Conclusion

When one partner develops dementia, both partners in a couple are facing illness-related stress and adaptive challenges. ICs may adopt a more supportive and care providing role, whereas PWDs end up at the receiving end. Intervention at an early stage is critical to prevent diverging roles and conflicting views to result in a deficient illness management as a couple and losses in individual wellbeing. An individual and dyadic burden is associated with high stress in ICs in particular, who often become the primary caregiver, simultaneously having to manage illness-related losses and emotions, as well as their own self-care and tasks. At the same time, the appreciation for emotional or practical support received from PWDs may start to erode within an asymmetrical role division and external social resources become less available to ICs. The extent of tasks and responsibilities taken over and away may feed into feelings of exclusion and insufficiency in PWDs. Recommendations for clinicians include consulting couples in how to communicate illness-related concerns, needs, and changes with each other and third parties, on the involvement of external resources and a mutually supported renegotiation of tasks and responsibilities in an early stage.

## Data availability statement

The raw data supporting the conclusions of this article will be made available by the authors, without undue reservation.

## Ethics statement

The studies involving human participants were reviewed and approved by Swiss Cantonal Ethics Committee (Basec-Nr. 2018–010907). The patients/participants provided their written informed consent to participate in this study.

## Author contributions

All authors listed have made a substantial, direct, and intellectual contribution to the work and approved it for publication.

## Conflict of interest

The authors declare that the research was conducted in the absence of any commercial or financial relationships that could be construed as a potential conflict of interest.

## Publisher’s note

All claims expressed in this article are solely those of the authors and do not necessarily represent those of their affiliated organizations, or those of the publisher, the editors and the reviewers. Any product that may be evaluated in this article, or claim that may be made by its manufacturer, is not guaranteed or endorsed by the publisher.
